# Prominent Human Health Impacts from Several Marine Microbes: History, Ecology, and Public Health Implications

**DOI:** 10.1155/2011/152815

**Published:** 2010-10-11

**Authors:** P. K. Bienfang, S. V. DeFelice, E. A. Laws, L. E. Brand, R. R. Bidigare, S. Christensen, H. Trapido-Rosenthal, T. K. Hemscheidt, D. J. McGillicuddy, D. M. Anderson, H. M. Solo-Gabriele, A. B. Boehm, L. C. Backer

**Affiliations:** ^1^Center for Oceans and Human Health, Pacific Research Center for Marine Biomedicine, School of Ocean and Earth Science and Technology, MSB no. 205, University of Hawaii, Honolulu, HI, 96822, USA; ^2^Rosenstiel School of Marine and Atmospheric Science, University of Miami, 4600 Rickenbacker Cswy, Miami, FL 33149, USA; ^3^Woods Hole Oceanographic Institution, Woods Hole, MA 02543, USA; ^4^Department of Civil, Architectural, and Environmental Engineering, University of Miami, Coral Gables, Florida and University of Miami Center for Oceans and Human Health, Key Biscayne, FL 33124-0630, USA; ^5^Department of Civil and Environmental Engineering, Stanford University, Stanford, California and University of Hawaii Center for Oceans and Human Health, Honolulu, HI 96822, USA; ^6^National Center for Environmental Health Centers for Disease Control and Prevention, 47770 Buford Highway NE MS F-46, Chamblee, GA 30341, USA

## Abstract

This paper overviews several examples of important public health impacts by marine microbes and directs readers to the extensive literature germane to these maladies. These examples include three types of dinoflagellates (*Gambierdiscus* spp., *Karenia brevis*, and *Alexandrium fundyense*), BMAA-producing cyanobacteria, and infectious microbes. The dinoflagellates are responsible for ciguatera fish poisoning, neurotoxic shellfish poisoning, and paralytic shellfish poisoning, respectively, that have plagued coastal populations over time. Research interest on the potential for marine cyanobacteria to contribute BMAA into human food supplies has been derived by BMAA's discovery in cycad seeds and subsequent implication as the putative cause of amyotrophic lateral sclerosis/parkinsonism dementia complex among the Chamorro people of Guam. Recent UPLC/MS analyses indicate that recent reports that BMAA is prolifically distributed among marine cyanobacteria at high concentrations may be due to analyte misidentification in the analytical protocols being applied for BMAA. Common infectious microbes (including enterovirus, norovirus, *Salmonella*, *Campylobacter*, *Shigella*, *Staphylococcus aureus*, *Cryptosporidium*, and *Giardia*) cause gastrointestinal and skin-related illness. These microbes can be introduced from external human and animal sources, or they can be indigenous to the marine environment.

## 1. Introduction

The health and welfare of humans residing in the coastal zone and in island communities are inextricably linked to the oceans and its foodwebs. The multitude of relationships between human societies and the oceans has led to many human dimensions of ocean issues and processes. The effects of climate change, pollution, population increases, and the myriad of anthropogenic effects attendant increasing population are all related in various ways to the microbial organisms that are at the base of marine ecosystems. In 2004, the National Science Foundation and the National Institutes of Environmental Health Sciences initiated collaborative funding of four centers for oceans and human health, and in the same year, the National Oceanic and Atmospheric Administration launched its Oceans and Human Health Initiative. One of the important accomplishments of these centers has been the cross-discipline synergistic collaboration of scientists within and between centers. This paper summarizes five areas of this research focusing on the oceans and human health.

## 2. *Gambierdiscus* spp. and Ciguatera Fish Poisoning

*Gambierdiscus* spp., a genus of dinoflagellates (division Phyrophyta), produce natural toxins that cause ciguatera fish poisoning (CFP) in humans. In contrast to other noxious dinoflagellates known for their dense “red tide” aggregations, *Gambierdiscus *do not form conspicuous blooms that color the water. These dinoflagellates normally grow epiphytically on various macroalgae in coral reef ecosystems within the 35°N–35°S latitudinal band. The dinoflagellates are consumed by herbivorous fish, beginning processes of bioaccumulation and biomodification through the reef food web, as the herbivores are consumed by carnivores and ultimately by humans. Several informative reviews on CFP may be found in Bienfang et al. [1], Dickey [2], and Dickey and Plakas [3] and references cited therein.

Ciguatera is a food-borne disease that has affected coastal populations and travelers in tropical and subtropical regions throughout the world for centuries. Ship logs as far back as the 16th century mention clinical symptomologies consistent with CFP [4, 5], and during WWII, CFP was a serious problem for military troops stationed in many Pacific island locals. There are at least 50,000 reported cases of CFP cases per year [6, 7], but due to the high degree of misdiagnosis and underreporting, it is estimated that the actual frequency of CFP cases is closer to 500,000 per year [8, 9]. It is estimated that >50% of the populations of small islands in the Caribbean and South Pacific have suffered from CFP; see reviews by Lewis [10], Lange [11], and Fleming et al. [12, 13]. 

Ciguatoxin produces gastrointestinal, neurological, and cardiovascular symptoms. These normally develop within 12–24 hours of eating contaminated fish. Gastrointestinal effects may disappear in four days. The normal progression of symptoms is gastrointestinal symptoms (e.g., diarrhea, abdominal pain, nausea, and vomiting), followed by neurological symptoms (e.g., numbness and tingling of hands and feet, dizziness, altered hot/cold perception, muscle aches, low heart rates, and low blood pressure). Symptoms may persist in some forms for weeks, months, or even years [9, 14–16]. Generally, feelings of weakness last ~1 week, and neurosensory manifestations (e.g., muscle aches, tingling extremities, and thermal reversals) commonly represent the most prolonged discomfort. The key pathonuemonic symptom is the neurological malady of reversal of hot/cold sensation. Fortunately, death is rare (i.e., <0.1%) and is most commonly the result of respiratory failure due to cardiovascular shock induced by severe dehydration. An interesting feature is that CFP intoxication does not confer any immunity in its victims, and to the contrary, its frequency results in heightened sensitivity to ciguatoxin.

The etiology of CFP was advanced when work in the Gambier Islands of French Polynesia by Yasumoto et al. [17–19] revealed that the guts of toxic (herbivorous) fish contained significant numbers of a dinoflagellate that was designated as a new genus and species known as *Gambierdiscus toxicus* [20]. Since then several new species have been added to the genus [21–23], and recent works using refined morphological and molecular sequencing techniques have caused substantial changes to the taxonomy of this genus [24, 25]. Derived from gambiertoxins produced by the dinoflagellates, ciguatoxin is a polar, lipid-soluble polyether. The toxin is heat stable, tasteless, odorless, and effective at extremely low (i.e., sub-ppb) concentrations; the severe analytical challenges presented by these properties have been central to the slow progress in detection of ciguatoxin for prevention and/or research purposes. 

A CDC report for a period in the 70's indicted that reported CFP incidences accounted for 25% of all food-borne outbreaks, which was five times the reported incidence for paralytic shellfish poisoning and neurological shellfish poisoning combined [26]. Kite-Powell [27] concluded that the economic impact from CFP exceeded that from any other form of hazardous algae bloom. Additionally, CFP is associated with societal/public health impacts in island communities due to dietary changes in response to concerns over the quality of local seafood. Chateau-Degat et al. [28] showed correlations of* Gambierdiscus* abundance and CFP with sea surface temperature in the South Pacific. This has attracted particular concern because warming oceans would expand the range of *Gambierdiscus* into higher latitudes where population density is generally greater. Though potentially influenced by improved awareness, the recognition of CFP in new geographic areas [29–31] has been suggested as evidence of an expanded range for CFP. Because of the underreporting artifact, it is difficult to ascertain whether CFP incidence is increasing over time, though expansion of international trade in seafood from tropical regions and climatic warming make this a distinct probability.

## 3. *Karenia brevis* and Neurotoxic Shellfish Poisoning

*Karenia brevis* is an unarmored photosynthetic dinoflagellate that lives primarily in the Gulf of Mexico and produces a suite of neurotoxins called “brevetoxins”. When *K. brevis* occurs at concentrations above ~100,000 cells/L, humans become aware of its presence of primarily as a result of three effects. At such concentrations, brevetoxins can cause: (1) fish kills, (2) filter-feeding shellfish to become extremely toxic to humans, and (3) respiratory distress, coughing, and eye irritation in humans due to aerosolization. Such impacts were experienced long before *K. brevis* was known to be the causative agent. Spanish explorers as early as the 15th century recorded fish kills in the Gulf of Mexico that were probably caused by *K. brevis*. Explorers also noticed that the native Americans were aware that shellfish could be toxic [32–34]. Davis [34] was the first to demonstrate that *K. brevis* was in fact the causative organism of fish kills and toxic shellfish. When first identified, it was named *Gymnodinium breve*. It was later renamed *Ptychodiscus brevis* and is currently known as *Karenia brevis.*

*K. brevis* is an unarmored dinoflagellate with a flattened shape, approximately 10–15 microns thick and 25–35 microns in diameter. It tends to swim up to the light during the day and disperse throughout the water column at night [35, 36]. It typically reproduces by binary fission every 2–5 days [37]. It is suspected to have a resting stage, perhaps in the benthos, but this is not firmly established at the present time [38]. 

Under typical nonbloom conditions, *K. brevis* concentrations are usually <10 cells/L offshore and ~1000 cells/L inshore [39]. For reasons that are still not well understood, it occasionally forms blooms with concentrations of one to tens millions of cells per liter. *K. brevis* has been observed sporadically throughout the Gulf of Mexico, but the largest and most frequent blooms occur along the west coast of Florida [32, 33, 40]. Although not as frequent, blooms also occur along the coast of Texas and Mexico [32]. About once a decade, conditions allow the Loop Current in the Gulf of Mexico to pick up blooms of *K. brevis* and transport it through the Straits of Florida and along the east coast of North America [32, 41]. As a result, blooms of *K. brevis* have been found as far north as North Carolina [41]. Currently, there is no evidence that these blooms along the east coast were generated locally, but rather were transported there from the Gulf of Mexico.

Along the west coast of Florida, where records have been kept for a half century, blooms of *K. brevis* are statistically more likely during the fall months, a month or two after the heaviest freshwater runoff from land [40] although they can occur at any time of the year. Huge year-to-year variations in bloom concentrations and/or duration are evident. In some years, essentially no blooms are observed, some years have short sporadic blooms, and some years large blooms may persist throughout the entire year.

Understanding the causes for the spatial and temporal occurrence/variability of *K. brevis* blooms is an area of active research. Factors that promote initiation of blooms may well be different from those that determine its extent and/or duration. Physical aggregation due the interaction of complex hydrography and the swimming behavior of *K. brevis* is probably also important. Olascoaga et al. [42] have argued that areas of low mixing due to certain hydrographic features along the west coast of Florida allow the slow growing *K. brevis* to accumulate large populations without being dispersed by mixing and dilution rates larger than its growth rate. 

A variety of hypotheses have been proposed for the sources of nutrients that would ultimately influence the concentrations and spatial extent of *K. brevis* blooms. Gunter et al. [43], Rounsefell and Nelson [44], Dixon and Steidinger [45], and Brand and Compton [40] have argued that nutrients in land runoff could be an important source. Lenes et al. [46], Walsh and Steidinger [47], and Walsh et al. [48] have argued that iron-rich atmospheric dust stimulation of nitrogen-fixing *Trichodesmium* in phosphorus-rich waters is an important nutrient source that can stimulate blooms of *K. brevis*. Hu et al. [49] have argued that nutrient-rich groundwater could be important. Vargo et al. [50] have argued that, along with other sources, fish that have died in blooms of *K. brevis* could be an important source of nutrients as they decompose. A perusal of the data on the spatial and temporal distribution of *K. brevis* blooms suggests that no one hypothesis will provide a simple explanation for its distribution. A variety of nutrient sources probably contribute to varying degrees to blooms of *K. brevis*.

*K. brevis* produces a suite of around 12 brevetoxins that activate the sodium channel of neurons [51]. At concentrations >100,0000 cells/L, blooms of *K. brevis* can kill many marine animals, including fish, turtles, sea birds, manatees, and dolphins [52]. Because brevetoxins are large lipid-soluble molecules, they tend to accumulate in fatty tissues and are not easily broken down or excreted. As a result, filter-feeding shellfish can accumulate high concentrations in their tissues. Brevetoxins are heat and acid stable, thus remain toxic after cooking. Human ingestion of toxic shellfish can lead to a variety of neurological and gastrointestinal symptoms [53], giving the general term “neurotoxic shellfish poisoning” (NSP). Because of this, government agencies in Florida monitor for *K. brevis *blooms and close shellfish beds to harvesting at times of blooms [53]. As a result, NSP is now rare, usually the result of illegal or uninformed harvesting of toxic shellfish.

Because the brevetoxins are lipid soluble, they also have the potential to accumulate and biomagnify up the food chain [54]. As a result, sublethal concentrations of *K. brevis* can still have lethal consequences [40]. Sublethal concentrations of *K. brevis* that do not kill nevertheless release brevetoxins that accumulate in organisms; thus higher level predators can accumulate high concentrations in their tissues. This may explain why dolphins and manatees have been found dead with high concentrations of brevetoxin in their stomachs and tissues in areas where no obvious blooms of *K. brevis* were observed [55]. Naar et al. [56] have found brevetoxins in the tissues of many fish species many months after the occurrence of a *K. brevis* bloom. These recent data suggest that brevetoxins may be more widespread in seafood than previously thought. 

As an unarmored dinoflagellate, *K. brevis* is delicate compared to most toxic dinoflagellate species. As a result, cells can be broken apart by turbulence due to wave action at the sea surface and along beaches. This results in aerosolization of brevetoxins that may be inhaled by humans and resulting in respiratory distress, coughing, and eye irritation in humans [57–60]. Hospital emergency room admittances for respiratory distress increase 50% when blooms of *K. brevis* occur [61] and is particularly acute in people with asthma [59, 62]. Beaches along the west coast of Florida are major recreational areas for Florida residents, including many elderly retirees and tourists. When large blooms of *K. brevis* develop, dead fish wash up on the beaches and/or brevetoxin aerosolization occurs and the tourism suffers considerable economic loss [63].

## 4. BMAA-Containing Cyanobacteria

Cyanobacteria have been a part of the human diet primarily in non-Western civilizations for centuries. Today, cyanobacteria are produced in mass, controlled cultivation processes and/or harvested from natural habitats and marketed as food supplements around the world. Historical and current uses of cyanobacteria and their derivative products are thoroughly reviewed by [64]. The amino acid *β*-N-methylamino-L-alanine (BMAA) is one of a number of biologically active natural compounds produced by cyanobacteria, and its potential importance in species of marine cyanobacteria has attracted considerable research attention recently. 

BMAA was originally discovered in cycad seeds [67], and later implicated the etiology of Amyotrophic Lateral Sclerosis/Parkinsonism Dementia Complex (ALS/PDC) that occurs among the Chamorro people of Guam [68]; see TemaNord [69] for an exhaustive review on this subject. BMAA is biomagnified in the Guam ecosystem and occurs in the brain tissues of Chamorros who died of ALS/PDC [70]. Axenic cultures of the endosymbiont cyanobacterium, *Nostoc *sp., isolated from the coralloid roots of the cycad palm, *Cycas micronesica *Hill, produce free BMAA at a concentration of 0.3 *μ*g *g* ^−1^. Following up on this work, Cox et al. [71] examined BMAA production in free-living and symbiotic clones representing the five morphotypes of cyanobacteria (cf. Rippka et al. [72].) A wide range of cyanobacterial strains were screened, including strains maintained at the University of Dundee in Scotland, Stockholm University in Sweden, and the University of Hawaii in the United States. Liquid chromatography-mass spectrometry (LC/MS) and high-performance liquid chromatography (HPLC) were employed to identify and quantify free and protein-associated BMAA for each sample. For free-living cyanobacteria, Cox et al. [71] found that BMAA was produced by members of all five cyanobacterial morphotypes as well as 95% of the genera and 97% of the strains that were screened. Analysis of *Nostoc *strains isolated from symbiotic relationships with fungi and host plants of broad taxonomic diversity indicated that 73% of these strains produced BMAA. The ubiquity of cyanobacteria in diverse terrestrial and aquatic environments suggests that ingestion of BMAA may occur through even less esoteric routes, including direct consumption of cyanobacteria or cyanobacterial hosts, bioaccumulation in additional food chains, or exposure to cyanobacteria-contaminated water supplies. Cox et al. [71] recommended that BMAA concentrations should be monitored in invertebrates, fish, and/or grazing animals used for human consumption that either directly consume cyanobacteria or forage on plants or prey that may have accumulated cyanobacteria-produced BMAA. This conclusion was reinforced by subsequent articles published in the Journal of the American Medical Association [73, 74] and Neuropathology and Applied Neurobiology [75].

**Figure 1:**
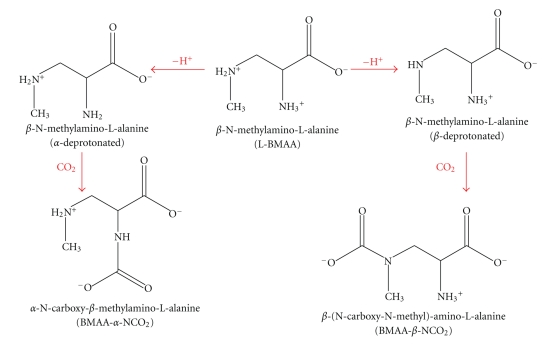
Reaction of the *α*- and *β*-amino groups of BMAA with CO_2_ to form the corresponding carbamate adducts (after [65, 66]).

The amino acid, *β*-N-methylamino-L-alanine (BMAA), is an excitotoxic neurotoxin that functions as a glutamate agonist. By virtue of its unique structural characteristics, BMMA reacts with CO_2_ at physiological pH to form *α*- and *β*-carbamate adducts [65, 66, 76] that are structurally similar to the neurotransmitter glutamate and its selective agonist N-methyl-d-aspartate (NMDA) (Figure 1). Rao et al. [77] have demonstrated that in the presence of 5% CO_2_, BMAA causes selective motor neuron loss in dissociated mixed spinal cord cultures at concentrations of ~30 *μ*M. These investigators also reported that the glutamate receptor antagonist 2,3-Dioxo-6-nitro-1,2,3,4-tetrahydrobenzo[f]quinoxaline-7-sulfonamide (NBQX) prevented BMAA-induced death, implicating BMAA in the excitotoxic activation of receptors for *α*-amino-3-hydroxy-5-methyl-4-isoxazolepropionic acid (AMPA) and kainic acid, and that BMAA selectively induced the production of reactive oxygen species (ROS) in motor neurons. More recently, Lobner et al. [78] demonstrated that BMAA also functions as an agonist for the NMDA and mGluR5 receptors in mouse cortical cell cultures. In addition to AMPA/kainate, NMDA and mGluR5 receptor activation and oxidative stress, BMAA may also induce toxicity by inhibiting the reuptake of glutamate. Furthermore, BMAA may be misidentified by transfer RNAs, resulting in its misincorporation into proteins.

Since the initial report of the widespread distribution of BMAA in representatives of all five cyanobacteria morphotypes by Cox et al. [71], there have been a number of conflicting studies published regarding the detection and quantification of BMAA in cyanobacteria (including blue green algae nutritional supplements). Eleven studies have confirmed the presence of BMAA in a wide range of marine, brackish, and freshwater cyanobacteria via LC/MS, LC/MS/MS or GC/MS methods that identify and detect underivatized [79, 80] and derivatized [75, 81–88] BMAA. By comparison, four studies were not able to detect the presence of BMAA in a wide range of cyanobacteria via HPLC, LC/MS, or LC/MS/MS that identify and detect derivatized [89] and underivatized BMAA [90–92]. These disparate findings are likely caused by analyte misidentification [91, 92] and/or differences in methodological sensitivities [88]. Because of potential coelution artifacts, we recommend that that BMAA identification and quantification be based on the LC/MS analysis of BMAA-specific fragments (*m/z* 88) [91] or AQC-derivatized BMAA (*m/z* 258) [88] in order to minimize the possibility of reporting false positive data [88, 91]. Based on this criterion, only four studies at the time of this writing have confirmed the presence of BMAA in cyanobacteria [79, 80, 87, 88]. Due to the serious implications of BMAA and neurodegenerative disease, it is further recommended that NMR analysis be used for the unequivocal identification of BMAA in biological samples. It should be noted that two studies employing LC/MS analysis of BMAA-specific fragments failed to detect BMAA in a wide range of cyanobacteria samples [91, 92]. We conclude that BMAA was either absent or below the limit of detection in these samples. Spáčil et al. [88] recommend subjecting samples to a pretreatment protocol to both remove impurities and to concentrate BMAA prior to LC/MS analysis. Finally, we recommend that future studies should monitor BMAA concentrations using only BMAA-specific LC/MS methods in animals used for human consumption that either directly consume cyanobacteria or forage on plants or prey that may have accumulated cyanobacteria-produced BMAA.

## 5. *Alexandrium fundyense* and Paralytic Shellfish Poisoning

Paralytic shellfish poisoning (PSP) has been recognized in the Pacific Northwest of the United States for centuries [93]. Human poisonings have been recorded primarily in North America, Asia, and Europe [94], but outbreaks have been reported worldwide [93, 95, 96]. PSP is caused by eating bivalve shellfish (clams, mussels, scallops, etc.) contaminated with one or more of a group of structurally related congeners of saxitoxin [97]. Filter-feeding fish can sometimes be vectors for the toxins as well. 

Saxitoxins are produced by dinoflagellates of the genera *Gymnodinium* [101], *Alexandrium* [95, 96], and *Pyrodinium* [97, 101]. These toxins act to selectively block the voltage-gated sodium channel of excitable membranes, thus blocking the generation and propagation of action potentials in nerve axons and skeletal muscle fibers. Mammals, birds, and fish can be affected by PSP toxins; however, humans are the most sensitive—the fatal oral dose of saxitoxin is 1–4 mg [102].

PSP symptoms begin to occur within 30 minutes to three hours of eating contaminated seafood. The initial symptoms include paresthesia and numbness around the lips and mouth [93]. These sensations then spread to the face and neck. Victims may also experience nausea and vomiting. In moderately severe poisonings, paresthesia progresses to the arms and legs. Victims may experience giddiness, incoherent speech, and light-headedness. In severe poisonings, death can result from respiratory failure and hypoxia. Historically, the fatality rate from PSP varies from no deaths in recent outbreaks in the U.S. or Europe to rates of 2%–14% in other parts of the world [93]. The frequency of mortalities is related to the availability of emergency hospital care, past experience with PSP outbreaks, and whether or not effective monitoring programs are in place to prevent contaminated shellfish from entering commercial markets. However, despite warning signs and other outreach efforts, recreational harvesters still become victims of PSP. For example, in June 2010, five cases of suspected PSP, including two fatalities, were reported in Anchorage Daily News due to shellfish collected from waters in Alaska, US. The victims had eaten personally harvested shellfish and crabs, including those from areas normally avoided because of historically high levels of contamination.

The causative organism in New England PSP outbreaks is *Alexandrium fundyense*. Although the Bay of Fundy and northeastern Canadian waters have a long history of PSP, in the U.S., toxicity was restricted to far-eastern Maine (ME) until 1972, when a massive, visible red tide of *A. fundyense* stretched from ME to Massachusetts (MA), causing toxicity in some southern areas for the first time. Virtually every year since 1972, western ME has experienced PSP outbreaks, and for the first 20 years of that interval, MA did as well. That pattern was a direct result of *A. fundyense* cysts being retained in western GOM waters after the 1972 bloom and subsequent events [103]. Between 1994 and 2004, toxicity was infrequent in MA and the southern GOM. Then in 2005, another massive bloom occurred [98], leading to closure of shellfish beds from ME to southern MA and 40,000 km^2^ of offshore federal waters as well. Economic losses in 2005 were estimated to be $50 million for the MA shellfish industry alone.

**Figure 2:**
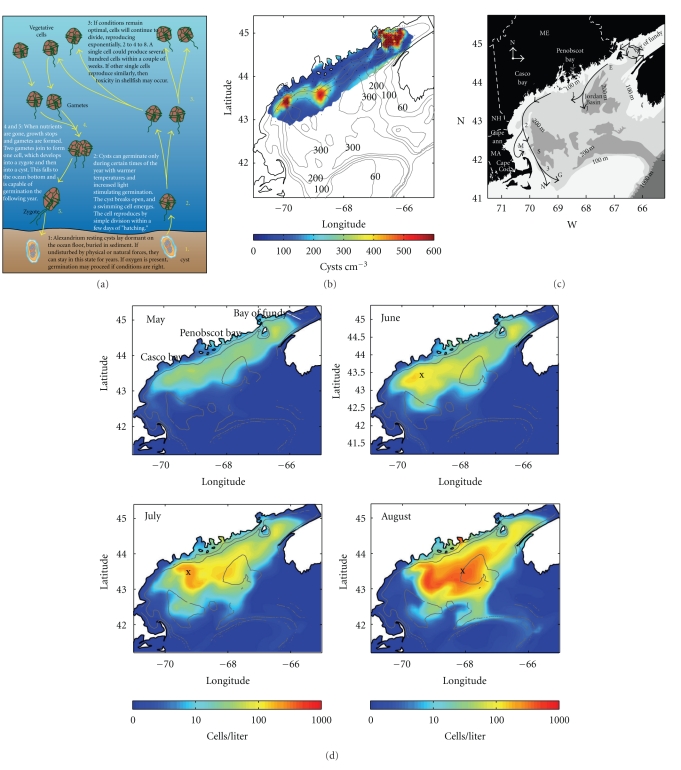
Upper left: life cycle of *A. fundyense*. Upper middle: distribution of cysts (number of cysts cm^−3^) in the upper 3cm of sediment derived from a 1997 survey of the Gulf of Maine [98] and surveys of the Bay of Fundy in 1981 (White and Lewis, 1982), 1982, and 1983 (data provided by Jennifer Martin, DFO). Upper right: schematic of the Maine Coastal Current, reprinted from McGillicuddy et al. [99]. Branch points are located at (1) Penobscot Bay, (2) Cape Ann, and (3) Great South Channel. Seven segments of the current are indicated: (E)astern, (J)ordan, (W)estern, (M)assachusetts, (S)tellwagen, (N)antucket, and (G)eorges Bank. Dashed white lines denote state boundaries of Maine (ME), New Hampshire (NH), and Massachusetts (MA). From Backer and McGillicuddy [100]. Reprinted with permission from *Oceanography*.

*A. fundyense* has a complex life cycle includes a resting cyst, a phase of vegetative growth, sexual reproduction, and re-encystment (Figure 2(a)). Observations indicate several salient characteristics of the vegetative cell distributions: patterns of abundance are gulf-wide in geographic scope; the distributions are associated with the Maine Coastal Current, and the center of mass of the distribution is from west to east during the April-to-August growing season [104]. This latter aspect is particularly notable given the coastal current flows in the opposite direction (Figure 2(b)). A model based on the seasonal mean flow that includes germination, growth, mortality, and nutrient limitation can produce simulations that are qualitatively consistent with the observations (Figure 2(c); [99]. In general, cells germinated from the major cyst beds in the Bay of Fundy and near Penobscot and Casco Bays (Figure 2(d)) are advected in the alongshore direction from east to west in the coastal current. Growth of the vegetative cells is limited primarily by temperature from April through June throughout the gulf whereas nutrient limitation occurs in July and August in the western gulf. Thus, the seasonal shift in the center of mass of cells from west to east can be explained by changing growth conditions: growth is more rapid in the western gulf early in the season due to warmer temperatures whereas growth is more rapid in the eastern gulf later in the season due to severe nutrient limitation in the western gulf during that time period. Hydrodynamic transport of these offshore populations to inshore shellfish beds is a key aspect regulating the PSP threat to human health [100, 105, 106].

In the wake of the historic bloom of 2005 in the western GOM, a suite of models was used to diagnose the underlying causes. Anderson et al. [98] described three factors to explain the 2005 bloom: (1) high abundance of resting cysts in fall 2004 that provided a large inoculums, (2) storms with strong northeast winds that carried toxic cells towards and along the coast, and (3) abundant fresh water runoff, providing macro- and micronutrients, a stratified water column, and an alongshore (towards the southwest) transport mechanism. These factors were evaluated using a sensitivity analysis that utilized field observations and a model of *A. fundyense* population dynamics [99, 107, 108], coupled to a regional circulation model to hindcast the 2005 bloom [109, 110]. Initial conditions of the three sensitivity experiments are identical to the central hindcast (an animated version of the central hindcast is available at http://science.whoi.edu/users/ruoying/Redtide_05/Papers/avg_fields.avi) in all respects except: experiment 1 utilizes the 1997 cyst map instead of 2004; experiment 2 is forced by winds from a more typical year (2004) instead of the strong downwelling-favorable winds of 2005; experiment 3 uses riverine discharge from a typical year (2004) instead of the anomalously large discharge of 2005. This sensitivity analysis suggests that high cyst abundance in the WGOM was the main cause of the 2005 bloom. Wind forcing was an important regulator, in the form of both episodic bursts of northeast winds and the downwelling-favorable mean condition, causing onshore advection of offshore populations. The anomalously high river runoff enhanced alongshore transport near the coast, but had limited impact on the gulf-wide bloom distribution. 

Model initial conditions are dependent on maps of *A. fundyense *cyst abundance obtained on an annual basis. Mathematical representations of laboratory-derived germination and growth data are used with these maps to drive the inoculation and development of the bloom. At this point, it is not yet possible to model the formation and deposition of new cysts from these blooms, though work is ongoing in this direction. This is an area where our lack of knowledge is evident—the termination of blooms remains poorly understood, and in particular, the relationship between bloom size and the size of the resulting cyst seedbed is not established, nor indeed is it intuitive. For example, some of the largest regional blooms (e.g., in 2005 [98]) were followed by very low cyst abundance on a regional basis whereas more modest bloom years (e.g., 2007) led to widespread and high density cyst accumulations {D.M.  Anderson, unpub.  data}. 

Modeling results to date suggest that simulations initiated from *A. fundyense* cyst distributions can capture large-scale seasonal patterns in the distribution and abundance of vegetative cells. To the extent that cyst abundance is a first-order predictor of regional bloom magnitude the following year in the WGOM (even though the converse is not true), that information can be used in a seasonal forecast of PSP on a regional basis. Near-real-time nowcasts and forecasts of harmful algal blooms (HABs) in the Gulf of Maine have been run routinely each year since 2006 (2006: http://science.whoi.edu/users/ruoying/Redtide_06/, 2007: http://omgrhe.meas.ncsu.edu/Redtide/Redtide_07/, 2008: http://omglnx3.meas.ncsu.edu/yli/08forecast/, 2009: http://omglnx3.meas.ncsu.edu/yli/09forecast/). During the bloom season, weekly updates have been made available to more than 150 managers and other officials and scientists involved with PSP outbreaks in the northeastern US. Web interfaces provide the latest model simulations, with one-week forecasts driven by meteorological predictions. At present, this kind of early warning appears to be the most practical approach to mitigating the impacts of these blooms, insofar as available direct intervention strategies are not practical by virtue of the fact that even in bloom conditions, *A. fundyense* is typically a small fraction of the total phytoplankton biomass.

## 6. Infectious Microbes

Waterborne infectious microbes normally include viruses, bacteria, and protozoa that can be transmitted through recreational exposure to seawater or consumption of seafood. Other groups of infectious microbes that are less commonly considered include the helminthes and yeasts. The infectious microbes differ from harmful algal species in that disease is caused by the growth of the microbes within humans. Thus, exposure to even low levels of infectious microbes can cause illness. Upon consumption, inhalation, or contact with the infectious microbe it then multiplies within the gastrointestinal system, respiratory tract, or within exposed skin resulting in human disease. Harmful algae, on the other hand, grow outside humans within external water bodies and release toxins that cause disease when the toxin-contaminated water is ingested or inhaled [62, 111]. Typically, the numbers of infectious microbes that are needed to cause disease are low; the precise number depends upon the virulence of the particular strain of the microbe [112] and also the immune status of the infected host. Risks to humans from infectious microbes are evaluated by considering human exposure to seawater or seafood in addition to the concentration of infectious microbes therein. Infectious microbes in seawater can be separated into two groups, those which are introduced from outside sources and those which are indigenous, hereafter referred to as allochthonous and autochthonous, respectively [113]. 

Infectious microbes in coastal waters impact the health of a large number of people globally. Worldwide, up to 170 million enteric and respiratory illnesses attributed to swimming in and consuming shellfish from infectious microbes in coastal waters [114]. In the US, 20,300 recreational beach advisories were reported in 2008 due to contamination with fecal bacteria, up from 6,200 in 1999 [115]. In the US, 33% of shellfish harvesting waters are impaired by infectious microbes [116]. In southern California alone, it is estimated that 1.5 million excess enteric illnesses occur per year from swimming in waters with infectious microbes, at a cost of $50 million per year [117]. One of the challenges of assessing the impact of infectious microbes on human health is that most illnesses associated with these infectious microbes are self-limiting, so medical advice is not always sought. In addition, identifying the etiologies of the illnesses can be challenging even in the most modern diagnostic laboratories. Most of the illnesses are also not reportable, so they are not tracked by a central agency. Yoder et al. [118] report *Cryptosporidium *as the most common etiology of freshwater recreational waterborne illness, and also reports *Vibrio* spp. as an important etiology for seawater-acquired recreational waterborne illness. A review of the epidemiology of seafood-associated illness in the United States between 1973 and 2006 [119] identifies *Vibrio parahaemolyticus* as being responsible for the most seafood illnesses (35%), norovirus and hepatitis A together responsible for the second highest number of illnesses (32%), and *Salmonella* and *Shigella* together responsible for the third highest number of illnesses (19%).

Introduced or allochthonous infectious microbes can come from human sewage, stormwater, feces of animals, and skin from infected humans during bathing. Allochthonous infectious microbes (from the virus, bacteria, and protozoan groups) include enterovirus, norovirus, *Salmonella*, *Campylobacter*, *Shigella*, *Cryptosporidium*, *Giardia*, *Legionella* sp. and *Staphylococcus aureus*. All of these microbes cause gastrointestinal disease with the exception of *Legionella* sp., which causes respiratory disease and *S. aureus* which causes skin disease. Transmission of helminthes via recreational contact with seawater is generally limited to developing countries and includes incidental ingestion of infective eggs or through contact with contaminated waters for forms that can penetrate skin [120]. In addition to infectious microbes from the virus, bacteria, and protozoan groups [121], coastal beach sands have also been implicated as a means of potentially transmitting pathogenic helminthes [122] and yeasts [123]. There are a number of excellent reviews on the occurrence of allochthonous infectious microbes, particularly bacteria, viruses, and protozoa, in coastal waters [124–126] and in shellfish [127, 128].  Table 1 provides examples of studies that report allochthonous pathogen occurrence in coastal waters. There are a limited number of studies that describe the fate and transport of allochthonous pathogens in the environment. These studies have generally highlighted the importance of pathogens associated with sediments [129] and show a positive correlation between the occurrence of pathogens and rainfall [130]. More research is clearly needed to understand the dynamics of pathogens once release to the environment.

*Vibrio* spp. represent the classic example of indigenous or autochthonous infectious microbes [144]. Other notable autochthonous infectious microbes include helminthes which are indigenous to fish populations which can be transmitted to humans via ingestion of undercooked fish [145] and amoeba which can enter the nasal cavity [118]. Common disease-causing *Vibrio* spp. include *V. cholerae* (toxigenic and nontoxigenic), *V. mimicus*, *V. parahaemolyticus*, *V. vulnificus*, and *V. alginolyticus*. *V. cholerae* and *V. mimicus* are closely related species, both of which cause gastroenteritis, *V. parahaemolyticus* causes gastroenteritis as well as wound infections, *V. vulnificus* can cause septis and wound infections, and *V. alginolyticus* can cause wound infections [119]. A number of studies have examined the environmental factors that control the occurrence of *Vibrio* spp. in coastal waters or within shellfish [146–149]. Common correlates to *Vibrio *concentrations include salinity and temperature; warmer temperatures typically correlate to higher *Vibrio* concentrations; salinity and *Vibrio *densities covary, but the direction depends on the organism. *Vibrio* have been shown in some cases to adsorb to zooplankton or phytoplankton and be associated with sediment [146, 150, 151]. Further information in *V. cholerae*, *V. parahaemolyticus*, and *V. vulnificus* can be found in excellent reviews on the organisms [152–154].

In order to protect human health and provide warnings for unsafe conditions, the presence of infectious microbes in coastal waters is evaluated through the use of “indicator” microbes. Indicator microbes are commensal inhabitants of the gastrointestinal tract of humans and are present in large numbers in fecal releases, especially releases from humans and warm blooded animals [155]. Indicator microbes are not necessarily pathogenic but are used as a surrogate for the presence of pathogenic microbes. For marine recreational waters, enterococci are the indicators recommended by the U.S. Environmental Protection Agency [156]. For freshwater recreational waters, both enterococci or *E. coli* are recommended [156]. The use of these microbes as indicators is supported by their correlation to measured, adverse, human health outcomes during epidemiology studies focused on illness during exposure to waters impacted by point sources of treated wastewater [157]. For shellfisheries in the US, fecal coliform are recommended to assess risk of exposure to pathogens through shellfish consumption [158]. 

Five basic dilemmas are associated with the use of indicator microbes to establish the safety of recreational waters. First, as enterococci and *E. coli* are natural inhabitants of the digestive tract of humans, the disease endpoint associated with these microbes is gastrointestinal disease. Although, gastrointestinal illness can be transmitted through water use [159], other types of illnesses can be transmitted during swimming including ear-, eye-, respiratory-, and skin-related diseases [160–162]. Second, studies have shown that in many cases indicator microbes do not track pathogenic microbes on a one-to-one basis [163, 164]. Ortega et al. [131] have shown that sites subject to sporadic increases in indicator levels are also characterized by detectable levels of pathogens, although the time that the pathogens are detected do not necessarily coincide with the time that the indicator microbe levels exceed regulatory guideline levels. Abdelzaher et al. [165] found that indicators and pathogens are generally elevated during similar environmental conditions (low solar radiation, after rainfall, and during a particular tidal period), but this correspondence was not always consistent. The third dilemma associated with the use of indicator microbes is the time required to measure indicator bacteria using traditional culture methods. Traditional culture methods require an 18 to 24 hours incubation period before detection of the bacteria. As a result, contaminated beaches can remain open for a significant period of time before levels are known, thereby resulting in exposures to human populations. Conversely, as contamination tends to be highly variable [166], beaches can also be closed during times when they are safe. The fourth dilemma is that the use of *E. coli* and enterococci to assess risk was established using data from epidemiology studies conducted at beaches polluted by point sources of treated wastewater. At the present time, most point sources of pollution in the developed world are well regulated and controlled to minimize human health impacts. Nonpoint sources, including urban and agricultural runoff, wildlife feces, bather shedding, and other “environmental reservoirs” are leading contributors of *E. coli* and enterococci to coastal waters. There is some evidence that there is a human health risk upon exposure to indicators from nonpoint pollution sources [167], but there still exists a great deal of debate on the topic [132]. Finally, fecal indicator bacteria cannot be used to protect individuals from exposure to autochthonous pathogens, like *Vibrio*. Significant risk of *Vibrio* infections could be present when there are no fecal bacteria. 

In the future, safety from infectious microbes in recreational waters and in seafood should consider multiple lines of evidence where indicator microbe measurements are supplemented with direct measures of a cluster of pathogenic targets that are relevant to the pollution sources and pathogens affecting the study area [168]. In addition, more work should be done to understand the ecology of allochthonous infectious microbes in the environment. Although these organisms are historically viewed as transient members of the microbial community of coastal waters, the coastal environment may serve as an important niche for these organisms to persist, exchange genetic material (for bacteria), and grow. Indeed, some researchers have found allochthonous pathogenic bacteria to be widespread in macroalgae of the Great Lakes [142]. Research is also needed to develop more rapid analyses methods for both indicators and pathogens [169], so that the time between measures and warnings can be reduced. Method detection limits also need to be improved if direct pathogen presence is to be considered for future monitoring purposes, as pathogens are typically present in very low numbers [169]. Currently, the procedures for measuring pathogens in environmental waters are time consuming, in part due to detection limit issues, and thus data on the environmental occurrence of infectious microbes is lacking. 

Ideally, the public should be warned prior to adverse water quality events. Given the current limitations in microbial measurements with respect to labor and time, early warning systems will likely rely on models [170, 171] designed to predict health risks based upon readily measureable environmental parameters and impending environmental conditions. Such models will require direct measures of microbial water quality for calibration and verification purposes. 

## Figures and Tables

**Table 1:** Examples of allothchonous human pathogens detected in coastal waters.

Viruses	Concentration/Occurrence	Reference
enteroviruses	Present in 9 of 72 1-liter samples using RT-PCR at Avalon Beach, CA*. Present in 1 of 18, 220-liter samples using culture based methods for brackish water in St. Lucie River Estuary, FL.	[131, 132]
adenoviruses	Present in 15 of 30 250-liter samples using PCR at Silver Beach, MI*	[133]
hepatitis A	105 to 30,771 viral particles/liter using Q-RTPCR at Imperial Beach, CA*	[134]
norovirus	2 of 19 samples in 110-liters using RT-PCR at Key West sites (FL)*	[135]
rotavirus (reovirus)	2 of 19 sites with 2–5 MPN/L at Italian coastline	[136]

Bacteria		

*Campylobacter*	Detected in 25 of 192 100–1000 mL Spanish marine recreational water samples using culture based methods	[137]
*Salmonella*	Detected in 70%–100% of samples from a lagoon in Brazil using culture-based methods, volume assayed not reported	[138]
*Staphylococcus*	60%–70% of approx. 100 mL seawater samples from Doheny and Avalon Beach, CA using culture-based methods. 37% of 668, 50 mL seawater samples from Hobie Cat Beach, FL using culture-based methods and confirmation by PCR	[139, 140]
Pathogenic *E. coli *	2 of 377 *E. coli* isolates from North Carolina and Southern California coastal waters using combined culture and PCR methods	[141]
*Shigella*	100% of algal mat samples from Lake Michigan near Burns Ditch by PCR	[142]
*Legionella sp.*	Found in 35 of 72 samples from Lake Pontchartrain with 1 of 72 positive for *L*. *pneumophila *	[140]

Protozoa		

*Cryptosporidium*	13.7 ± 1.7 oocysts/L on weekends at Chesapeake Bay beach, MD	[143]
*Giardia*	9.1 ± 1.1 cysts/L on weekends at Chesapeake Bay beach, MD	[143]

*Volumes reported do not account for the fact that a fraction of water sample was used during polymerase chain reaction (PCR), reverse-transcriptase- (RT-) PCR, or quantitative (Q) PCR.
